# Modulation of Myostatin/Hepatocyte Growth Factor Balance by Different Hemodialysis Modalities

**DOI:** 10.1155/2017/7635459

**Published:** 2017-03-28

**Authors:** Pasquale Esposito, Edoardo La Porta, Marta Calatroni, Maria Antonietta Grignano, Samantha Milanesi, Daniela Verzola, Yuri Battaglia, Marilena Gregorini, Carmelo Libetta, Giacomo Garibotto, Teresa Rampino

**Affiliations:** ^1^Department of Nephrology, Dialysis and Transplantation, Fondazione IRCCS Policlinico San Matteo and University of Pavia, Pavia, Italy; ^2^Department of Internal Medicine, Istituto Nazionale per la Ricerca sul Cancro, University of Genoa and IRCCS Azienda Ospedaliera Universitaria San Martino-IST, Genoa, Italy; ^3^Nephrology and Dialysis Unit, St. Anna University Hospital, Ferrara, Italy

## Abstract

*Background.* In this study we investigated the relevance of myostatin and Hepatocyte Growth Factor (HGF) in patients undergoing hemodialysis HD and the influence of different HD modalities on their levels.* Methods.* We performed a prospective crossover study in which HD patients were randomized to undergo 3-month treatment periods with bicarbonate hemodialysis (BHD) followed by online hemodiafiltration (HDF). Clinical data, laboratory parameters, and myostatin and HGF serum levels were collected and compared.* Results.* Ten patients and six controls (C) were evaluated. In any experimental condition myostatin and HGF levels were higher in HD than in C. At enrollment and after BHD there were not significant correlations, whereas at the end of the HDF treatment period myostatin and HGF were inversely correlated (*r*  −0.65, *p* < 0.05), myostatin serum levels inversely correlated with transferrin (*r*  −0.73, *p* < 0.05), and HGF levels that resulted positively correlated with BMI (*r* 0.67, *p* < 0.05). Moving from BHD to HDF, clinical and laboratory parameters were unchanged, as well as serum HGF, whereas myostatin levels significantly decreased (6.3 ± 4.1 versus 4.3 ± 3.1 ng/ml, *p* < 0.05).* Conclusions.* Modulation of myostatin levels and myostatin/HGF balance by the use of different HD modalities might represent a novel approach to the prevention and treatment of HD-related muscle wasting syndrome.

## 1. Introduction

Patients suffering from chronic kidney disease (CKD), mainly those undergoing hemodialysis (HD), often present malnutrition and muscle wasting syndrome, which directly correlate with morbidity and mortality [1]. Several mechanisms have been involved in the regulation of energy and muscular homeostasis, including cytokines and molecules with systemic and paracrine action (the so-called “myokines”) and intracellular signalling pathways, that may have positive or negative effects on muscle growth, also affecting muscle response to injury through the inhibition or stimulation of muscle stem cells (“satellite cells”) [2]. The Growth Differentiation Factor-8 (GDF-8)/myostatin, a member of TGF-*β* family, is primarily expressed in skeletal muscle and has the effect of limiting muscle growth. It also circulates in the blood and acts on muscle tissue, binding a cell-bound receptor called Activin type 2B [3]. The importance of myostatin was emphasized by several studies, demonstrating its increase in muscular atrophy and chronic disease [4–6].

The Hepatocyte Growth Factor (HGF) is another key factor in the regulation of skeletal muscle homeostasis. HGF was firstly recognized as a product of mesenchymal cells with an action predominantly addressed to stimulate growth, motility, and differentiation of epithelial-derived cells [7]. Subsequently, it has been demonstrated that HGF is also present in muscle tissue and is essential for the response to cellular damage and the repair of damaged tissue, inducing activation and rapid cell division of muscle satellite cells [8]. In CKD patients an upregulation of myostatin gene expression in skeletal muscle has been found, which was strictly related to IL-6 expression, suggesting a link between myostatin and microinflammation [9]. Moreover, it has been demonstrated in the clinical setting that myostatin serum levels in HD patients are elevated and inversely related to muscle strength [10].

Similarly, also HGF levels appear to be influenced by uremia and HD. Indeed, it has been proved that HD causes a prompt and prolonged release of HGF into the circulation, mainly because of the leukocyte activation associated with HD treatment [11].

Therefore, considering their elevated circulating levels and apparently opposite effects on muscular metabolism, it is conceivable that myostatin and HGF accumulation, as well as their balance, may be important in the pathogenesis of malnutrition and muscle wasting syndrome in uremic subjects.

This is the reason why we decided to investigate the relevance of myostatin and HGF in HD patients and whether their serum levels could be modulated by using different HD modalities.

## 2. Patients and Methods

We performed a prospective 6-month crossover study, enrolling clinically stable uremic patients undergoing standard bicarbonate hemodialysis (BHD) at least for 6 months. Patients with acute infections, active immunological diseases, immunosuppressive therapy, previous transplantation, or history of malignancies were excluded from the study. Sex-matched healthy subjects were the control group. Patients were randomized into two groups according to a 2 × 2 crossover design. Group 1 was treated with BHD for the first 3 months and then switched to online hemodiafiltration (HDF) for additional 3 months; group 2 was initially treated with HDF and then with BHD.

BHD was performed with cellulose diacetate membranes (DICEA, Baxter Healthcare, Baxter, Iowa, USA) with a blood flow of 300–350 ml/min and a dialysate flow of 600 ml/min. In HDF, the replacement volume was standardized to 25–30% of the total treated blood volume, using a high-flux Helixone membrane (FX100, Fresenius Medical Care, Bad Homburg, Germany).

At the beginning of the study (enrollment phase) and at the end of each experimental period we evaluated anthropometric and nutritional parameters, including body mass index (BMI), predialysis serum levels of phosphate, albumin, transferrin, lymphocyte count, and blood urea nitrogen, and dialysis adequacy (evaluated as single pool KT/V-spKT/V). In each patient, before the hemodialysis session, serum was withdrawn and tested for HGF and myostatin levels by ELISA (Quantikine; R&D Systems, Minneapolis, MN, USA; detection limit 40 pg/ml and 5.3 pg/ml, resp.), assuming those samples presenting values under the detection limit as zero.

The study was conducted in accordance with the Declaration of Helsinki and was approved by the Ethics Committee of the Fondazione IRCCS Policlinico San Matteo of Pavia, Italy. Written informed consent was obtained from each participant prior to enrollment in the study.

### 2.1. Statistical Analysis

Quantitative variables were represented by mean ± standard deviation (SD) or interquartile ranges (IQR) if they were not normally distributed (Shapiro Test).

Differences among control subjects and HD patients in different experimental conditions were assessed by analysis of variance (ANOVA), Student's *t*-test, or nonparametric Mann–Whitney test when appropriated. In order to evaluate a possible carry-over effect, we compared the variations of myostatin and HGF levels between subjects of group 1 (i.e., starting with BHD) versus group 2 (i.e., starting with HDF). Correlations among myostatin, HGF levels, and variables were analyzed with Spearman-Rho. All tests were two-sided and *p* < 0.05 was considered statistically significant. Data analysis was performed with GraphPad Prism statistical package (version 5.00, GraphPad Software, San Diego, California, USA).

## 3. Results

### 3.1. Patient Characteristics

We enrolled ten patients (65.5 ± 13.1 years, seven males) with a dialysis vintage of 70.8 ± 18 months.

Mean BMI was 28.4 ± 4.7 kg/m^2^; three patients were diabetic. At the time of enrollment all patients were undergoing thrice-weekly 4-hour BHD, with spKT/V of 1.46 ± 0.4.

Six healthy subjects (48.1 ± 12.7 years, four males; BMI 26.5 ± 2.3 kg/m^2^, *p* = 0.3 versus patients) constituted the control group (C). After randomization, five patients (50%) underwent BHD as initial treatment (group 1) and five received HDF (group 2).

### 3.2. Myokine Profile

At enrollment and during the two different experimental conditions (BHD or HDF) myostatin levels in patients resulted higher than in C, without reaching statistical significance (at enrollment 6 ± 3.4 versus 3.1 ± 0.6 ng/ml, resp., *p* = 0.07).

On the opposite, HGF resulted under the detection limit in C and four HD patients, reaching levels of 150.5 pg/ml (IQR 44.2–681) in the remaining six HD patients.

The correlation analysis performed on data collected at each experimental phase showed that, at enrollment and the end of BHD period, myostatin levels did not correlate with any clinical (age, dialytic vintage, and BMI) and laboratory (albumin, transferrin, phosphorus, and calcium) parameter. On the contrary, myostatin levels measured at the end of HDF period resulted in being inversely correlated with transferrin (*r* = −0.73, *p* < 0.05) and HGF levels (*r* = −0.65, *p* < 0.05).

Interestingly, similar results were obtained for HGF levels. Indeed, while HGF did not correlate with any variable at the enrollment and after BHD, it resulted in being positively correlated with BMI (*r* = 0.67, *p* < 0.05) at the end of HDF treatment.

### 3.3. Effect of Different Dialytic Treatments

During the two different treatment periods, there were no significant changes in clinical and laboratory parameters (Table 1).

**Figure 1:**
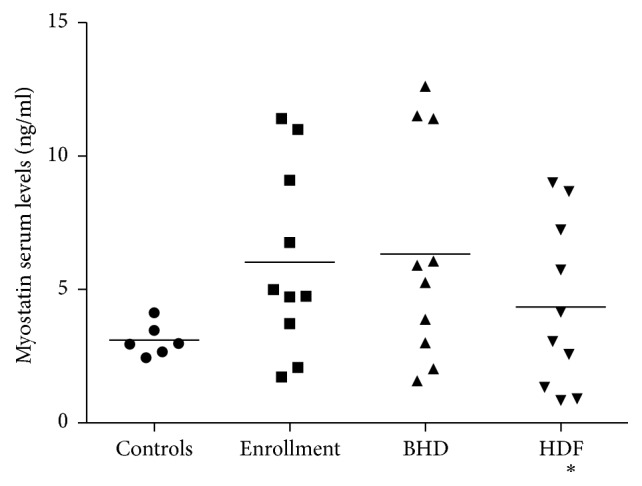
Effect of different dialysis modalities on myostatin serum levels. BHD = bicarbonate hemodialysis; HDF = online hemodiafiltration. At the enrollment all the patients were on BDH for at least 6 months. ^*∗*^*p* < 0.05 versus enrollment and BHD.

After shifting from BHD to HDF, HGF levels remained unchanged [BHD 93 (IQR 42–231.8) versus HDF 156.7 (IQR 88–201) pg/ml, *p* = 0.2], whereas serum myostatin levels significantly decreased [BHD 6.3 ± 4.1 versus HDF 4.3 ± 3.1 ng/ml, *p* = 0.001 (Figure 1)]. The differences in myostatin levels from BHD to HDF were −1.4 ± 1.7 ng/ml for group 1 and −1.1 ± 2.3 ng/ml for group 2, *p* = 0.9.

Therefore, starting with BHD or HDF did not seem to have a significant impact on HD modality-induced changes in myostatin levels.

## 4. **Discussion**

With this study we have demonstrated that in regular HD patients the use of different HD modalities could have different effects on myokine balance. In particular online HDF, a convective dialytic technique associated with better depuration capacity and a higher rate of cytokine removal, significantly reduced myostatin levels, without affecting HGF [12].

This is an important consideration since myostatin and HGF seem to exert opposite effects on the modulation of muscle cell metabolism. In fact, myostatin, where serum levels are elevated in CKD and in cachectic states, may act as a negative regulator of muscle growth and differentiation [13, 14], whereas HGF exerts proliferative and differentiative stimuli on muscle cells. So, it is possible that these molecules play a role in the pathogenesis of muscle wasting in HD, also by a feedback mechanism. However, our findings highlighted that only in patients undergoing online HDF myostatin and HGF resulted in being inversely correlated. Similarly, we found that only during HDF myostatin and HGF revealed a relationship with nutritional parameters. In particular, myostatin, as expected by its role as negative metabolic regulator, was inversely related to transferrin, a parameter strongly associated with a good nutritional status [15]. On the opposite, HGF was directly related to BMI, which, in turn, seems to be protective in HD patients [16].

Therefore, it seems that high-volume online HDF treatment could be able to restore a more “physiological” condition in which myostatin and HGF are counterregulated and correlated with nutritional parameters.

This preliminary finding, which surely needs further confirmations, together with the evidence that BHD and HDF exert different effects on myostatin and HGF levels, might have a great clinical relevance. Indeed, in the past years research activity has been addressed to the development of new dialysis modalities, mainly convective techniques, which could improve clearance of uremic toxins, also providing higher clearances of middle molecules, such as inflammatory cytokines [17].

In this view, high-volume online HDF, using high biocompatible membranes and ultrapure dialysate, has been found to be more efficient than standard BHD, resulting in being also associated with a lower mortality rate [18–20]. Our data provide new and intriguing evidence on the potential advantages of convective strategies, showing that the use of HDF, through the modulation of myokine levels, might also promote a state of better muscle trophism, as previously only hypothesized [21]. This is particularly relevant considering that, in spite of several efforts to understand the underlying mechanisms of these high-risk conditions, currently there are no established strategies to prevent and manage malnutrition and wasting syndrome in HD.

On the other hand, there is a growing interest in the study of myokines, in particular myostatin, as therapeutic targets for the treatment of skeletal muscle wasting/atrophy under diverse clinical settings, including denervation, AIDS, cancer, diabetes, and chronic heart failure [22]. Indeed, several experimental studies demonstrated that pharmacological inhibition of myostatin by genetic interference or specifically designed antibody was associated with increased muscle mass and improved metabolic profile [23, 24], suggesting that myokine modulation might realistically represent a promising approach to prevent and treat muscle wasting.

We are aware that our data present some weaknesses. In fact, although the crossover design of our study was intended to reduce interindividual heterogeneity, since each patient served as his own control, the small number of patients enrolled constitutes a limitation of this study.

Moreover, we did not perform a structured assessment of the nutritional status of our patients, also considering that the treatment periods were probably too short to appreciate any significant clinical change in muscle mass or strength.

## 5. Conclusions

Malnutrition and muscle wasting syndrome remain unsolved problems in HD patients, strictly related to the elevated morbidity and mortality distinctive of this patient population.

Therefore, the understanding of underlying mechanisms could provide new therapeutic targets and strategies to face these conditions. With the preliminary findings of our study we offer a new point of view on this issue, showing that the use of different HD modalities, in particular convective-based techniques, can influence myokine profile.

In light of this evidence, we hypothesize that HD-mediated myokine modulation might potentially represent a novel approach to the prevention and treatment of HD-related muscle wasting syndrome. Prospective studies with a larger number of patients are needed to confirm these data and evaluate their clinical impact.

## Figures and Tables

**Table 1:** Laboratory and dialysis parameters in patients at enrollment and at the end of each treatment period with different dialysis modalities.

	Enrollment	BHD	HDF	*p*
BMI (kg/m^2^)	28.4 ± 4.7	28.8 ± 4.8	28.9 ± 5.1	0.5
Serum albumin (g/dl)	3.8 ± 0.3	3.7 ± 0.2	3.8 ± 0.2	0.5
C-RP (mg/dl)	0.47 (9.3–0.67)	0.6 (0.3–1)	0.45 (0.3–0.77)	0.3
Transferrin (mg/dl)	157.8 ± 15.1	167.7 ± 42.3	172.2 ± 32.1	0.2
Phosphate (mg/dl)	4.4 (3.8–5.7)	4.7 (4.3–5.3)	4.1 (3.9–4.7)	0.5
Calcium (mg/dl)	9 ± 0.3	9.3 ± 0.6	9.2 ± 0.5	0.6
PTH (pg/ml)	210 (149–359)	173.9 (106–599)	227 (156–361)	0.3
Lymphocyte count	1283 ± 673	1255 ± 588	1396 ± 567	0.5
BUN (mg/dl)	71.4 ± 8.6	80.2 ± 18.5	77.2 ± 13.1	0.4
spKT/V	1.46 ± 0.4	1.36 ± 0.25	1.42 ± 0.2	0.4
Predialysis myostatin (ng/ml)	6.0 ± 3.4	6.3 ± 4.1	4.3 ± 3.1	0.001^*∗*^
Number of patients	10	10	10	
Predialysis HGF (pg/ml)	150.5 (44.2–681)	93 (42–231.8)	156.7 (88–201)	0.2
Number of patients	6	6	7	

Data are expressed as mean SD or IQR (25–75).

BHD = bicarbonate hemodialysis; HDF = online hemodiafiltration; C-RP = C-reactive protein; BUN = blood urea nitrogen; spKT/V = single-pool KT/V; HGF = hepatocyte growth factor.

^*∗*^HDF versus BHD.
